# Fast recovery of cardiac function in PIMS-TS patients early using intravenous anti-IL-1 treatment

**DOI:** 10.1186/s13054-021-03548-y

**Published:** 2021-04-07

**Authors:** Maria Vincenza Mastrolia, Edoardo Marrani, Giovanni Battista Calabri, Manuela L’Erario, Ilaria Maccora, Silvia Favilli, Pier Paolo Duchini, Ilaria Pagnini, Gabriele Simonini

**Affiliations:** 1 Rheumatology Unit, Meyer Children’s University Hospital, Viale Gaetano Pieraccini, 24, 50139 Firenze, Italy; 2 Cardiologic Unit, Meyer Children’s University Hospital, Firenze, Italy; 3 Pediatric Intensive Care Unit, Meyer Children’s University Hospital, Firenze, Italy; 4 Rheumatology Unit, NEUROFARBA Department, Meyer Children’s University Hospital, University of Florence, Firenze, Italy

**Keywords:** PMIS-TS, Kawasaki disease, COVID 19, SARS CoV-2 infection

**To the editor**,

We read with interest the manuscript entitled “Anakinra treatment in critically ill COVID-19 patients: a prospective cohort study” by Kooistra et al. [1] reporting the potential efficacy of anakinra (ANA) to control the hyperinflammation in COVID-19 patients.

In our clinical practice, we adopted the early use of intravenous ANA for the treatment of cardiac disfunction in Pediatric Inflammatory Multisystem Syndrome temporally associated with SARS CoV-2 infection (PIMS-TS) patients. During the second COVID-19 wave, 9 PIMS-TS children were admitted to Meyer Children’s University Hospital in Florence (mean age of 10.2 y [IQR] 8.5–13). Echocardiography revealed a left ventricular ejection fraction (LVEF) ≤ 40% in 5/9 patients. In these 5 children, ANA was adopted as first-line therapy and administered as continuous intravenous infusion at 10 mg/kg/day (400 mg/day maximum dose). Within the first day of ANA therapy, fractionated IVIG (2 g/kg) and intravenous steroids (one methylprednisolone pulses [30 mg/kg/day, maximum 1 g/day] in 3 consecutive days followed by 1 mg/kg/day intravenous methylprednisolone) were subsequently associated. At median time of 24 h (range 12–36 h) from starting ANA, all patients restored LVEF to > 55% along with a progressive reduction of troponin and N-terminal pro B-type natriuretic peptide (NT pro-BNP) values (Fig. 1). A concomitant reduction until discontinuation of inotropic support was obtained together with the recovery of clinical sings and inflammatory parameters.

**Fig. 1:**
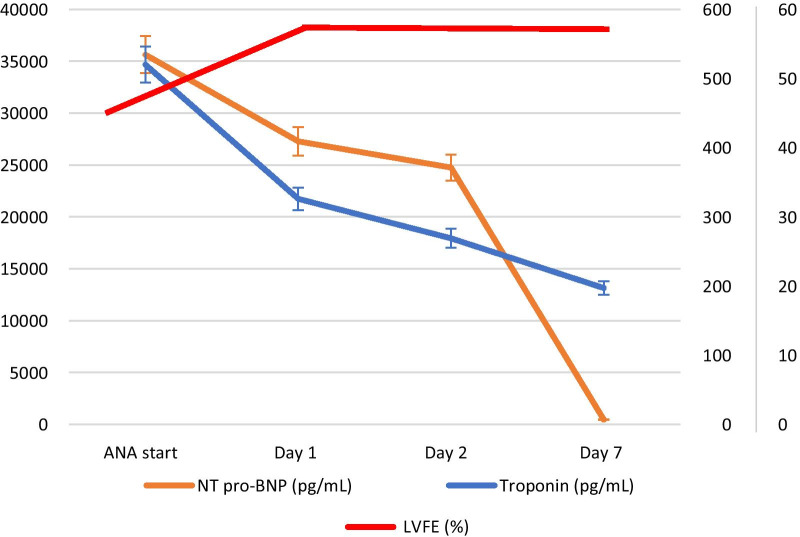
Trend of mean NT pro-BNP and troponin values in relation to PIMS-TS patients’ LVEF after starting anakinra (ANA)

In order to prevent the inflammatory rebound, ANA therapy was tapered in 2 weeks, then switched subcutaneously and stopped after 5 weeks (range 4–6).

One month after discharge, echocardiography reported stably normal findings.

The early use of ANA prompted a rapid and sub-stained LEVF improvement over one day from admission. Our results further support the assumption that an aggressive, early and overtime immunomodulatory approach in PIMS-TS patients with myocardial involvement may induce a faster time to recovery, as quickly damping the cytokine storm [2, 3]. However, the cumulative effect of ANA in combination with subsequent IVIG and steroid use could be advocated as effective in restoring a normal LVEF. Due to the poor peripheral perfusion and hemodynamic instability into the early phases of PIMS-TS, continuous intravenous infusion may be the preferable administration route. Subcutaneous injections might be considered as maintenance therapy after achieving stable conditions [3].

Future randomized controlled trials and long-term follow-up could test the hypothesis that a step-down immunomodulatory approach could be preferred in PIMS-TS patients experiencing myocardial disfunction to avoid a further progression and/or the onset of sequalae over time.

## Data Availability

The complete clinical reports of each patient are available for the reviewers if requested.
