# Supplementary material for: Fast recovery of cardiac function in PIMS-TS patients early using intravenous anti-IL-1 treatment
Source: Crit Care. 2021 Apr 7;25:131. doi: 10.1186/s13054-021-03548-y (PMC8024939; doi:10.1186/s13054-021-03548-y)
Supplement: Related texts

# Author’s reply

**Authors:** Emma J. Kooistra, Matthijs Kox, Peter Pickkers

**Affiliations:** 5 grid.10417.33 0000 0004 0444 9382 Department of Intensive Care Medicine, Radboud University Medical Center, 6500 HB Nijmegen, The Netherlands; 6 grid.10417.33 0000 0004 0444 9382 Radboud Center for Infectious Diseases, Radboud University Medical Center, 6500 HB Nijmegen, The Netherlands

## Anakinra to treat COVID-19-related hyperinflammation

We thank Mastrolia and colleagues for their letter in response to our study. They describe the use of anakinra in 5 pediatric patients suffering from Pediatric Inflammatory Multisystem Syndrome temporally associated with Severe Acute Respiratory Syndrome Coronavirus 2 (SARS-CoV-2) infection (PIMS-TS) and a left ventricular ejection fraction (LVEF) ≤ 40%. Within 36 h, both LVEF and cardiac biomarkers recovered, enabling discontinuation of inotropic support in all patients.

Although severe pediatric cases of SARS-CoV-2 infection are rare, children with PIMS-TS present with a complex multisystem inflammatory syndrome which often necessitates admission to the Intensive Care Unit (ICU). This syndrome is distinct from Coronavirus Disease 2019 (COVID-19), not only in terms of clinical features, but also because PIMS-TS may manifest up to weeks after SARS-CoV-2 infection [4]. Cardiac function, as in the 5 cases reported by Mastrolia et al., is commonly affected in patients presenting with PIMS-TS [5]. Furthermore, preclinical data have shown beneficial effects of anakinra in Kawasaki disease [6], which shares many features with PIMS-TS, which may have prompted Mastrolia et al. to initiate this therapy.

While the results presented by Mastrolia and colleagues are encouraging, several aspects clearly limit the conclusions. First, no control group receiving standard care was included. This is of relevance, as previous reports describe that LVEF may also recover fairly quickly without anakinra treatment [5]. Second, the authors used anakinra in combination with high-dose methylprednisolone and intravenous immunoglobulins. Especially the use of high dosages of corticosteroids impedes proper assessment of the effects of anakinra. Third, anakinra treatment was tapered over a period of 5 weeks, to prevent an inflammatory rebound according to the authors. In our study in critically ill COVID-19 patients [1], anakinra treatment was not tapered. Inspired by the strategy of Mastrolia et al., we assessed whether cessation of anakinra treatment resulted in an increase in inflammatory parameters in our COVID-19 patients. We analyzed our data until 8 days after cessation of anakinra, which, given the half-life of 4–6 h, should reveal a rebound effect, if present. As depicted in Fig. 2 however, no such inflammatory rebound effect was observed.

**Fig. 2:** Plasma levels of **a** C-reactive protein, **b** procalcitonin, **c** white blood cell counts, **d** ferritin, and **e** body temperature on day of admission to the intensive care unit (ICU), start of anakinra therapy, and until 8 days following cessation of anakinra. Results of 13 anakinra-treated patients of which data were available on multiple days after cessation of anakinra treatment are shown. Data are presented as geometric mean with 95% confidence intervals. Anakinra therapy was initiated 12 [7–17] and ceased 22 [18–25] days following ICU admission (median [IQR]). P-values were calculated using paired t-tests and mixed effect model analysis on log-transformed data. No inflammatory rebound effect was observed following cessation of anakinra therapy

Taken together, the interesting findings by Mastrolia and colleagues certainly warrant further study in a more controlled setting. Our results indicate that, in adult COVID-19 patients, a rebound inflammatory response does not occur when anakinra is stopped, indicating that tapering of anakinra treatment is not necessary in this patient category.
